# Permissive Parenting Style and Anemia Are Associated with Developmental Delays Among Under-Five Children in Bandung District, West Java, Indonesia

**DOI:** 10.3390/children13070856

**Published:** 2026-06-26

**Authors:** Cynthia Angeline, Rahmat Budi Kuswiyanto, Sri Endah Rahayuningsih, Rodman Tarigan, Diah Asri Wulandari, Susi Susanah

**Affiliations:** Department of Child Health, Faculty of Medicine, Universitas Padjadjaran, Dr. Hasan Sadikin General Hospital, Bandung 40161, Indonesia; cynthia22001@mail.unpad.ac.id (C.A.); rahmat.budi.kuswiyanto@unpad.ac.id (R.B.K.); sri.endah@unpad.ac.id (S.E.R.); rodman.tarigan@unpad.ac.id (R.T.); diah.asri@unpad.ac.id (D.A.W.)

**Keywords:** anemia, developmental delay, early childhood, parenting style

## Abstract

**Highlights:**

**What are the main findings?**

- Anemia affected 20.2% of children under five years of age and was particularly common among those younger than 24 months.
- Permissive parenting was associated with a higher likelihood of anemia, and children with anemia were more likely to experience developmental delays.

**What are the implications of the main findings?**

- Public health interventions targeting childhood anemia must expand beyond nutritional support to integrate comprehensive parental education focusing on optimal parenting practices.
- Early developmental screening and aggressive hemoglobin monitoring must be prioritized at the primary healthcare level, specifically targeting the critical window of children under 24 months of age to prevent long-term developmental delays.

**Abstract:**

**Background/Objectives:** Anemia in early childhood remains a key global health issue due to its impact on growth and development. While biological determinants of anemia have been extensively studied, parenting styles remain unexplored. This study aimed to examine the association between parenting styles, anemia, and developmental outcomes among under-five children. **Methods**: From February to March 2026, a cross-sectional study was carried out in Bandung, Indonesia, involving children aged 6–59 months who visited the Pasirkaliki Primary Health Centre. Anemia was confirmed by laboratory testing, defined as a hemoglobin level ≤ 11 g/dL. The Parenting Styles and Dimensions Questionnaire was used to assess parenting styles, while the Pre-Screening Developmental Questionnaire was used to examine child development. Statistical analyses were performed using the Mann–Whitney U test, Chi-square test or Fisher’s exact test, and logistic regression analysis. **Results:** One hundred and ninety-three subjects were included in the analysis, among which 20.2% were anemic, with a significantly higher proportion among children aged below 24 months (*p* < 0.001). Permissive parenting was significantly more common among children with anemia and was associated with higher odds of anemia (aOR = 10.31; 95% CI: 3.92–27.10). Children with anemia had significantly higher odds of developmental delay (aOR = 19.49; 95% CI: 6.46–58.84), after adjustment for child age, maternal education, and family income. **Conclusions:** Permissive parenting was associated with anemia, while anemia was associated with increased odds of developmental delay in under-five children, highlighting the importance of considering not only biological but also psychosocial factors in early child health interventions.

## 1. Introduction

The first five years of life represent a critical period for long-term developmental outcomes, during which the brain undergoes rapid growth and high neuroplasticity [1,2]. During this phase, both genetic and environmental factors play essential roles in child development. Adequate nutrition, responsive caregiving, appropriate parenting practices, early stimulation, and a supportive environment are key determinants of optimal child growth and development [2,3].

Developmental delay is defined as the failure to achieve age-appropriate developmental milestones [4]. This is still a major issue in public health on a global scale. Around 43% of children under the age of five in low- and middle-income nations are at danger of not attaining their whole developmental potential, according to the World Health Organization (WHO) [5,6]. The stimulation, detection, and early intervention of child development integrated into primary healthcare services is one of Indonesia’s national programs that implements early detection and intervention strategies [3].

Among environmental determinants, parenting style plays a central role in influencing child development. Parenting refers to the consistent patterns of attitudes and behaviors adopted by parents in raising their children [7,8]. According to Baumrind’s classification, parenting styles are categorized into authoritative, authoritarian, and permissive [9]. These parenting practices influence not only psychosocial development but also children’s nutritional intake and overall health outcomes. Evidence suggests that appropriate feeding practices are associated with improved nutritional status and better micronutrient profiles, including iron status. Furthermore, parental education and counseling have been shown to improve hemoglobin levels in children, highlighting the importance of caregiving behavior in preventing anemia [10,11].

Anemia in children is still a big issue in public health across the globe. About 40% of children globally and 38.4% in Indonesia suffer from this condition [12,13]. Reports showed that among children aged 6–24 months in Bandung, anemia was found in 40.7% of subjects, with the majority of cases (87.3%) caused by iron deficiency anemia [14]. Iron deficiency during early life may result in long-term effects on growth and neurodevelopment [15,16]. Several neurological processes, including as myelination, neurogenesis, and neuronal development, rely on iron, which is essential for erythropoiesis [17].

Previous studies have demonstrated that iron deficiency is associated with impairments in cognitive, motor, language, and behavioral development [18]. Nutritional status, including iron status, is therefore closely linked to developmental outcomes [19]. In addition, parenting practices are important determinants of child nutrition, as they influence dietary intake, health-seeking behavior, and exposure to infections. Poor nutritional status may compromise immune function and increase susceptibility to infections, thereby elevating the risk of anemia. These findings highlight the need for a comprehensive approach that integrates clinical management with family-based interventions, including nutrition education and improved parenting practices [20].

Despite growing evidence on the individual roles of parenting and anemia, previous studies have mostly examined these factors separately. Moreover, findings on the association between anemia and developmental outcomes remain inconsistent across studies, suggesting the need for a more integrative approach. Evidence simultaneously investigating the interaction between parenting styles, anemia status, and developmental outcomes remains limited, particularly in primary healthcare settings in Indonesia.

Because child development is influenced by complex interactions between biological and psychosocial determinants, understanding these relationships in an integrated framework is essential. This study aimed to analyze the association between parenting styles, anemia, and developmental outcomes among under-five children.

## 2. Materials and Methods

### 2.1. Study Design and Setting

This cross-sectional analytical study was conducted at Pasirkaliki Primary Health Centre and its associated Integrated Healthcare Centres in Bandung, West Java, Indonesia. The study was carried out between February and March 2026.

### 2.2. Participants

**Figure 1:**
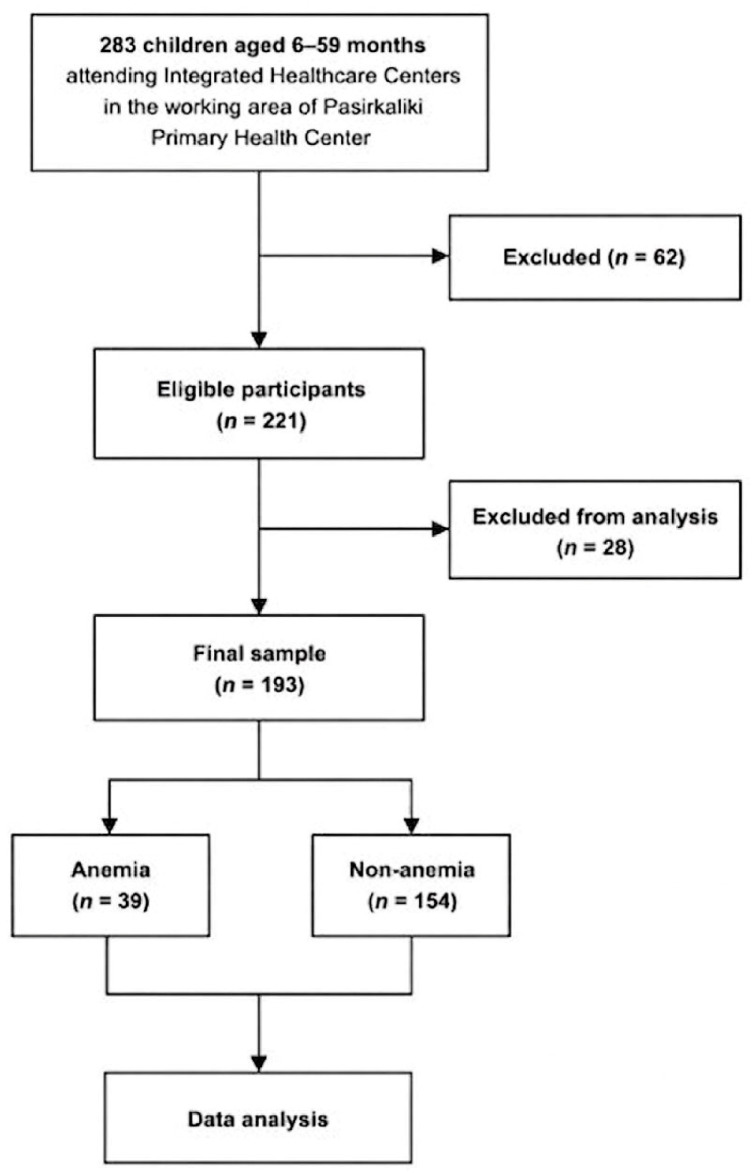
Flow diagram of participant selection.

Children aged 6–59 months who attended the Pasirkaliki Primary Health Centre or its associated Integrated Healthcare Centres during the study period and underwent anemia assessment through physical examination and laboratory testing were eligible for inclusion. Children with severe or chronic illnesses, cerebral palsy, epilepsy, developmental or mental disorders, a history of blood transfusion, hematological disorders, or malignancies were excluded. Participants were recruited using a consecutive sampling method. A total of 193 children were included in the final analysis (Figure 1).

This study was approved by the Research Ethics Committee of Universitas Padjadjaran (Approval No. 124/UN6.KEP/EC/2026). Written informed consent was obtained from all parents prior to enrollment.

### 2.3. Data Collection and Measurements

Data were collected through structured interviews with parents, as well as physical examinations, developmental assessments, and laboratory evaluations of the children. Information on maternal characteristics, including age, education, occupation, and family income, as well as child characteristics, including sex, age, gestational age, nutritional status, birth weight, and use of the Maternal and Child Health Handbook, was obtained during visits to the Primary Health Centre or its associated Integrated Healthcare Centres for routine growth monitoring and immunization services. Physical examinations, developmental assessments, and laboratory evaluations were performed on the children by medical doctors who had received training in standardized study procedures.

Parenting style was assessed using the short-form Indonesian version of the Parenting Styles and Dimensions Questionnaire (PSDQ), which consists of 32 items representing three parenting dimensions. This culturally adapted instrument has demonstrated acceptable validity and reliability among Indonesian parents, with a reported Cronbach’s alpha of 0.70 [21,22]. Anthropometric measurements were subsequently obtained to determine nutritional status according to the World Health Organization (WHO) Child Growth Standards. Child development was assessed using the Developmental Pre-screening Questionnaire (KPSP), with a score ≤ 6 indicating developmental delay [3].

Hematological assessment was performed during the study visit. Venous blood samples were collected by trained nurses and stored in standardized cooled containers for transport to the Hasan Sadikin Hospital Laboratory. Transportation time ranged from 30 min to 1 h before the samples reached the laboratory facility. Complete blood count (CBC) analysis was performed using an automated hematology analyzer (Sysmex XN-1000, Sysmex Corporation, Kobe, Japan), which provided measurements of hemoglobin (Hb) level, red blood cell (RBC) count, reticulocyte hemoglobin equivalent (Ret-He), and erythrocyte indices, including mean corpuscular volume (MCV), mean corpuscular hemoglobin (MCH), and mean corpuscular hemoglobin concentration (MCHC).

To further characterize anemia and assess potential iron deficiency, additional parameters were evaluated among children diagnosed with anemia, including Ret-He, the Mentzer index, microcytic–hypochromic erythrocyte morphology, and serum ferritin level. For biochemical assessment, serum ferritin levels were measured using a fully automated immunoassay analyzer (Alinity i, Abbott Laboratories, Abbott Park, IL, USA).

Anemia was defined according to the criteria for children aged 6–59 months as a Hb level < 11.0 g/dL. Anemia severity was further classified as mild (10.0–10.9 g/dL), moderate (7.0–9.9 g/dL), or severe (<7.0 g/dL) [23]. Iron deficiency anemia is characterized by microcytic–hypochromic anemia (MCV < 80 fL, MCH < 27 pg) and serum ferritin levels < 12 ng/mL [12,24]. Reticulocyte hemoglobin equivalent (Ret-He) < 27.65 pg was defined according to previous studies [14].

### 2.4. Statistical Analysis

Data were analyzed using IBM SPSS Statistics for Windows, Version 27.0 (IBM Corp., Armonk, NY, USA) and logistic regression modelling. Descriptive statistics were used to summarize subject characteristics and laboratory findings. Numerical variables were presented as mean, standard deviation (SD), minimum and maximum value for the age-stratified hematological and iron status table. Categorical variables were presented as frequencies and percentages. Normality of numerical variables was assessed using the Shapiro–Wilk test. Comparisons between anemia and non-anemia groups were performed using the Chi-square test or Fisher’s exact test for categorical variables and the Mann–Whitney U test for numerical variables, as appropriate.

Bivariate logistic regression was used to estimate crude prevalence odds ratios (PORs) and 95% confidence intervals (CIs). To address potential confounding, multivariable logistic regression was performed to estimate adjusted odds ratios (aORs). Two multivariable logistic regression models were constructed. In the first model, anemia status was the dependent variable with parenting style as the main exposure. In the second model, developmental status was the dependent variable with anemia status as the main exposure. Both models were adjusted for child age group, maternal education, and family income. Statistical significance was set at *p* < 0.05.

## 3. Results

### 3.1. Participant Characteristics

A total of 283 children aged 6–59 months attended the Integrated Healthcare Centers during the study period. After applying the inclusion and exclusion criteria, 221 children were deemed eligible. Of these, 28 were excluded due to technical issues (21 clotted blood samples and 7 unsuccessful blood collections), resulting in a final sample of 193 children, consisting of 95 females (49.2%) and 98 males (50.8%).

The participants’ ages ranged from 20.0 to 48.0 months, with a median age of 35.0 months. The median ages of the children with anemia were 21.0 months (IQR: 12.0–37.0) and 40.0 months (IQR: 24.0–49.0), which is a statistically significant difference (*p* = 0.001). Upon age group stratification, a notable disparity in distribution was discovered between the two categories (*p* < 0.001). The largest proportion of children with anemia was observed in the 13–24-month age group, whereas most non-anemic children (72.7%) were in the 25–59-month age group.

In both the anemia and non-anemia groups, the majority of participants were born at a gestational age of ≥37 weeks (92.3% vs. 93.5%), had a birth weight of >2500 g (94.9% vs. 92.9%), and had normal nutritional status. The proportion of participants reporting complete utilization of the Maternal and Child Health handbook was comparable between the anemia and non-anemia groups (64.1% vs. 64.9%), with no significant difference between the groups.

Maternal characteristics were also similar across groups. The median maternal age was 32.0 years (IQR: 29.0–35.0) in the anemia group and 31.5 years (IQR: 28.0–36.0) in the non-anemia group. Most mothers in both groups had a middle-level education (high school/vocational), and the majority were homemakers. Additionally, most families had a monthly income ≤ the regional minimum wage, accounting for 79.5% in the anemia group and 81.2% in the non-anemia group. Baseline characteristics of the study subjects are presented in Table 1.

### 3.2. Anemia Status, Severity, and Hematological Profiles Among Children

Based on WHO classification of anemia, 39 of 193 children (20.2%) were classified as anemic and 154 (79.8%) as non-anemic. Among the anemic children, 24 (61.5%) had mild anemia and 15 (38.5%) had moderate anemia. No cases of severe anemia were identified.

To provide deeper insights into the hematological characteristics of the study population, the parameters were further stratified by both age groups and anemia status, as summarized in Table 2. The mean hemoglobin values were 9.96 ± 1.11 g/dL in children aged 6–12 months, 9.85 ± 0.73 g/dL in those aged 13–24 months, and 10.26 ± 0.57 g/dL in those aged 25–59 months. Microcytic–hypochromic anemia was observed in 38 of 39 anemic children (97.4%). Ret-He < 27.65 pg was observed in 25 of 39 anemic children (64.1%), with the Mentzer index > 13 in 35 of 39 (89.7%). Serum ferritin was measured in all 39 children with anemia, but valid results were available for 29 children due to clotting in 10 samples, with serum ferritin < 12 ng/mL observed in 11 of 29 children (37.9%).

### 3.3. Parenting Style, Anemia, and Developmental Status

Permissive parenting was significantly more common among children with anemia than among non-anemic children (46.2% vs. 7.8%; *p* < 0.001), whereas authoritative parenting was more frequent among non-anemic children (77.9% vs. 43.6%) (Table 3). In bivariate logistic regression with anemia status as the outcome, permissive parenting was associated with higher odds of anemia compared with authoritative parenting (POR = 10.59 (4.35–25.78); *p* < 0.001). Authoritarian parenting was not significantly associated with anemia (POR = 1.28 (0.39–4.18); *p* = 0.699). After adjustment for child age group, maternal education, and family income, permissive parenting remained independently associated with anemia (aOR = 10.31 (3.92–27.10); *p* < 0.001), whereas authoritarian parenting remained non-significant (aOR = 1.46 (0.43–4.98); *p* = 0.545).

The direction of analysis for developmental outcomes was specified a priori with anemia as the exposure and developmental delay as the outcome. Developmental delay was observed in 17 of 39 children with anemia (43.6%) and in 8 of 154 children without anemia (5.2%) (Table 4). In bivariate logistic regression, children with anemia had higher odds of developmental delay than non-anemic children (POR = 14.10 (5.44–36.55); *p* < 0.001). After adjustment for child age group, maternal education, and family income, anemia remained independently associated with developmental delay (aOR = 19.49 (6.46–58.84); *p* < 0.001). The complete parameters of the multivariate logistic regression model are detailed in Table 5.

## 4. Discussion

Low hemoglobin level or low red blood cell count is the hallmark of anemia [25]. Because of its long-term effects on growth and development, childhood anemia remains a major public health concern. Anemia affected 20.2% of under-five children in the present study. However, the cases were dominated by the 6–24-month age group, which made up 61.5% of the total burden. This finding is consistent with Susanah et al. (2022), who reported a high prevalence of 40.7% in the same age group [14]. Similar findings have been reported elsewhere. Fentaw et al. (2023) showed that anemia was more common among children aged 6–11 months and 12–23 months [26], while Li et al. (2025) also found a higher prevalence of anemia among younger children [27]. Together, these findings suggest that children in the first two years of life are particularly vulnerable to anemia. During this phase of rapid growth, iron stores accumulated during the prenatal period naturally begin to deplete. The transition to complementary feeding (MPASI) often fails to bridge this nutritional gap [2,28].

In this study, male children slightly predominant in the study population (50.8% vs. 49.2%). However, a higher proportion of anemia cases was observed among females (56.4% vs. 43.6%). Williams et al. (2024) reported only slight variations are observed of anemia prevalence in early childhood [29].

The high proportion of normal nutritional status among children with anemia, particularly in those under 24 months (24 of 39 subjects), can be explained by the concept of hidden hunger. This condition occurs when energy and macronutrient intake is sufficient to support normal growth, but micronutrient intake especially iron during the complementary feeding period is inadequate. Consequently, children may appear nutritionally normal based on clinical assessment, yet still present with anemia on laboratory examination [30].

In the Indonesian context, this issue is closely related to MPASI. The SEANUTS II Indonesia study reported that complementary feeding is characterized by low dietary diversity, with a high reliance on staple foods such as rice, cereals, and noodles, while intake of animal-source foods remains limited. This leads to inadequate iron intake and contributes to a high prevalence of anemia in early childhood [31]. Similarly, previous studies have shown that complementary foods in Indonesia are often low in iron density despite adequate energy intake [32]. As a result, children may appear to have normal growth while still experiencing hidden micronutrient deficiencies, particularly iron deficiency anemia.

In contrast, no significant associations were observed between anemia and gestational age, nutritional status, birth weight, use of the Maternal and Child Health handbook, maternal education, maternal occupation, or family income. Although these findings appear inconsistent with several previous studies [27,33,34], which have shown that prematurity, lower maternal education, and lower socioeconomic status are associated with an increased likelihood of anemia, they likely reflect the relative homogeneity of the study population. The participants were recruited from a relatively uniform urban community in Bandung, where most families shared similar socioeconomic backgrounds and comparable maternal education levels.

A key contribution of this study lies in the identification of permissive parenting as a strong correlate of anemia. After adjustment for child age, maternal education, and family income, permissive parenting remained independently associated with anemia (aOR = 10.31; 95% CI: 3.92–27.10). Children exposed to permissive parenting exhibited markedly higher odds of anemia compared to those raised under authoritative parenting. While the relationship between parenting style and anemia has been underexplored, our findings provide empirical support for the hypothesis that caregiving environments play a critical role in shaping child health outcomes. These findings are consistent with previous studies emphasizing the important role of caregivers in shaping feeding practices [35,36].

Permissive parenting is characterized by low control, limited structure, and reduced parental enforcement of routines, including feeding practices [37]. Such environments may facilitate irregular eating patterns, selective feeding, and insufficient intake of iron-rich foods, ultimately increasing the likelihood of anemia. Thus, the observed association may be linked to suboptimal feeding behaviors and reduced parental engagement in nutritional regulation.

This study demonstrates a strong association between anemia and developmental delay. Multivariable logistic regression was performed to adjust for child age group, maternal education, and family income. After adjustment, anemia remained independently associated with developmental delay (aOR = 19.49; 95% CI: 6.46–58.84). Children with anemia were significantly more likely to exhibit delayed development, underscoring the biological plausibility of this relationship.

The findings showed that the predominant type of anemia was microcytic–hypochromic anemia. The presence of microcytic–hypochromic anemia should prompt further investigation of its underlying etiology, which commonly includes iron deficiency anemia (IDA), anemia of chronic disease, infection-related anemia, and thalassemia. In this study, IDA was confirmed in 11 of 39 anemic children (28.2%) based on serum ferritin levels < 12 ng/mL. In addition to iron deficiency, anemia may also occur due to infections or chronic diseases. A study by Susanah et al. (2025) reported that anemia in children with stunting and tuberculosis was found in 23.9% of cases [38].

Furthermore, in interpreting microcytic and hypochromic anemia in this population, it is important to consider the high burden of hemoglobinopathies in Indonesia, which lies within the global thalassemia belt [39]. In this study, advanced red blood cell indices were used to help differentiate these conditions. Although the mean Mentzer index across anemic subgroups remained above 13, individual-level analysis identified 27 children across the entire study population with a Mentzer index below 13, 4 of whom were within the anemic subgroups. Crucially, when cross-referenced with the Shine and Lal Index (SLI), 25 of these 27 children (including all four individuals in the anemic subgroups) presented an SLI value below 1530, strongly reinforcing the probability of an underlying thalassemia trait or hemoglobinopathy variant rather than pure nutritional iron deficiency. However, as iron deficiency and thalassemia traits may coexist and certain cases may present with overlapping hematological indices, definitive differentiation requires confirmatory hemoglobin electrophoresis or genetic testing.

Iron plays a crucial role in brain development, including myelination, neurotransmitter synthesis, and energy metabolism [17]. Reduced oxygen-carrying capacity due to anemia may impair cerebral oxygenation, particularly in the developing brain, which has disproportionately high metabolic demands [40]. These findings are consistent with an expanding body of evidence demonstrating that iron deficiency anemia is associated with adverse cognitive, motor, and behavioral outcomes in early childhood.

Importantly, the coexistence of permissive parenting, anemia, and developmental delay observed in this study suggests a potentially interconnected pathway involving both biological and psychosocial mechanisms. This highlights a critical gap in current child health strategies, which often focus on biological factors without adequately considering the caregiving context.

From a clinical and public health perspective, these results have important implications. First early identification and management of anemia should remain a priority. Second, interventions aimed at improving child nutrition should extend beyond supplementation and dietary recommendations to include parental education on structured and responsive feeding practices.

The limitations of this study include that it used a cross-sectional design, which cannot robustly test for causal relationships. Some potential confounding factors, such as detailed feeding practices, were not comprehensively assessed and may have influenced the observed associations. In addition, although screening indices (Mentzer Index and Shine and Lal Index) were used to identify children with possible thalassemia traits or other hemoglobinopathies, confirmatory diagnostic tests such as hemoglobin electrophoresis or molecular genetic analysis were not performed. Consequently, the potential presence of undiagnosed hemoglobinopathies may have contributed to the observed anemia burden and could not be fully distinguished from iron deficiency anemia. Furthermore, reliance on self-reported questionnaires to assess parenting style introduces the potential for information bias, particularly recall bias and social desirability bias, which may affect the accuracy of the findings. Therefore, the results should be interpreted with caution.

Future research employing longitudinal or prospective cohort designs is warranted to clarify causal pathways. Further studies should also examine feeding practices as potential mediators to better elucidate the mechanisms linking parenting style to anemia and developmental outcomes. In addition, future investigations should incorporate confirmatory hemoglobinopathy testing to better distinguish nutritional anemia from inherited red blood cell disorders in populations where thalassemia and other hemoglobin variants are prevalent.

## 5. Conclusions

This study found that 20.2% of children aged 6–59 months had anemia, most of which was mild to moderate and characterized by a microcytic–hypochromic pattern. Permissive parenting was associated with anemia after adjustment for child age, maternal education, and family income. In addition, anemia was associated with developmental delay. These findings highlight the importance of integrating early anemia detection and appropriate management with parenting-focused interventions to improve child health and developmental outcomes. However, given the cross-sectional nature of the study, longitudinal research is needed to confirm the underlying causal relationships.

## Figures and Tables

**Table 1:** Subject characteristics.

Variables	Total (*n* = 193)	Anemia (*n* = 39)	Non-Anemia (*n* = 154)	*p*-Value
Sex, *n* (%)				0.315 ^a^
Male	98 (50.8)	17 (43.6)	81 (52.6)	
Female	95 (49.2)	22 (56.4)	73 (47.4)	
Age (months)	35.0 (20.0–48.0)	21.0 (12.0–37.0)	40.0 (24.0–49.0)	0.001 ^c^*
Age group, *n* (%)				<0.001 ^a^*
6–12 months	31 (16.1)	10 (25.6)	21 (13.6)	
13–24 months	35 (18.1)	14 (35.9)	21 (13.6)	
25–59 months	127 (65.8)	15 (38.5)	112 (72.7)	
Gestational age (weeks)	38.0 (38.0–39.0)	38.0 (37.0–39.0)	38.0 (38.0–38.3)	0.547 ^c^
Gestational age, *n* (%)				0.728 ^b^
<37 weeks	13 (6.7)	3 (7.7)	10 (6.5)	
≥37 weeks	180 (93.3)	36 (92.3)	144 (93.5)	
Nutritional status (SD)	−0.27 (−0.98–0.36)	−0.43 (−1.02–0.30)	−0.19 (−0.97–0.42)	0.250 ^c^
Nutritional status, *n* (%)				0.287 ^a^
Normal	177 (91.7)	38 (97.4)	139 (90.3)	
Overweight	8 (4.1)	0 (0.0)	8 (5.2)	
Underweight	8 (4.1)	1 (2.6)	7 (4.5)	
Birth weight (g)	3000 (2800–3400)	3100 (2800–3300)	3000 (2800–3400)	0.928 ^c^
Birth weight, *n* (%)				1.000 ^b^
>2500 g	180 (93.3)	37 (94.9)	143 (92.9)	
1600–2500 g	13 (6.7)	2 (5.1)	11 (7.1)	
Maternal and child health handbook utilization, *n* (%)				0.923 ^a^
Complete	125 (64.8)	25 (64.1)	100 (64.9)	
Incomplete	68 (35.2)	14 (35.9)	54 (35.1)	
Maternal age (years)	32.0 (28.0–35.0)	32.0 (29.0–35.0)	31.5 (28.0–36.0)	0.750 ^c^
Maternal education, *n* (%)				0.733 ^a^
Low (≤Junior High School)	50 (25.9)	12 (30.8)	38 (24.7)	
Middle (Senior High/Vocational)	126 (65.3)	24 (61.5)	102 (66.2)	
High (≥Diploma)	17 (8.8)	3 (7.7)	14 (9.1)	
Maternal occupation, *n* (%)				0.612 ^a^
Civil servant (teacher/midwife)	2 (1.0)	1 (2.6)	1 (0.6)	
Private employee	16 (8.3)	3 (7.7)	13 (8.4)	
Self-employed	6 (3.1)	2 (5.1)	4 (2.6)	
Homemaker	169 (87.6)	33 (84.6)	136 (88.3)	
Family income, *n* (%)				0.812 ^a^
≤UMK	156 (80.8)	31 (79.5)	125 (81.2)	
>UMK	37 (19.2)	8 (20.5)	29 (18.8)	

Notes: Categorical data are presented as *n* (%), and numerical data are presented as median. Regional minimum wage is based on 2025 Bandung City Minimum Wage (UMK) of Rp 4,482,914.09. ^a^ Chi-square test; ^b^ Fisher’s exact test; ^c^ Mann–Whitney test; * statistically significant (*p* < 0.05).

**Table 2:** Hematological parameters by age group and anemia status.

Age Group	Parameter	Anemia (*n* = 39)			Non-Anemia (*n* = 154)		
		*n*	Mean ± SD	Min–Max	*n*	Mean ± SD	Min–Max
6–12 months	Hb	10	9.96 ± 1.11	7.20–10.70	21	12.15 ± 0.82	11.10–14.20
	MCV	10	73.98 ± 3.53	67.30–78.40	21	72.54 ± 3.95	65.60–78.90
	MCH	10	23.26 ± 1.57	20.80–25.20	21	23.40 ± 1.75	19.90–26.50
	Ret-He	10	26.06 ± 2.30	22.00–28.50	21	27.43 ± 2.05	22.90–30.40
	MI	10	17.33 ± 2.68	13.20–23.00	21	13.99 ± 1.50	11.40–16.50
	SF	6	26.12 ± 14.09	15.30–51.40			
13–24 months	Hb	14	9.85 ± 0.73	8.40–10.90	21	11.76 ± 0.57	11.10–13.10
	MCV	14	72.46 ± 7.09	57.10–79.30	21	73.97 ± 5.71	60.20–83.10
	MCH	14	22.77 ± 3.28	16.00–26.90	21	24.01 ± 2.63	16.40–27.30
	Ret-He	14	26.08 ± 4.77	17.60–33.00	21	27.29 ± 3.60	16.60–31.70
	MI	14	16.02 ± 3.28	9.80–21.00	21	15.21 ± 2.84	8.50–20.00
	SF	10	27.56 ± 37.86	4.90–126.30			
25–59 months	Hb	15	10.26 ± 0.57	9.10–10.90	112	12.58 ± 0.91	11.00–16.20
	MCV	15	70.71 ± 7.25	54.30–80.10	112	76.31 ± 4.08	61.10–85.60
	MCH	15	22.67 ± 3.02	16.00–26.40	112	25.49 ± 1.62	19.40–28.30
	Ret-He	15	25.83 ± 4.00	17.30–29.60	112	29.08 ± 2.17	21.40–32.60
	MI	15	15.47 ± 3.29	9.00–20.00	112	15.49 ± 1.81	10.00–19.90
	SF	13	22.58 ± 22.55	5.20–90.50			

Hb: Hemoglobin (g/dL); MCV: Mean Corpuscular Volume (fl); MCH: Mean Corpuscular Hemoglobin (pg); Ret-He: Reticulocyte Hemoglobin Equivalent (pg); MI: Mentzer Index; SF: Serum Ferritin (ng/mL).

**Table 3:** Association between parenting style and anemia status among under-five children.

Exposure Variable	Anemia *n* (%)	Non-anemia *n* (%)	Crude POR (95% CI)	Adjusted OR (95% CI)	*p*-Value for aOR
Parenting style					
Authoritative	17 (43.6)	120 (77.9)	Reference	Reference	—
Authoritarian	4 (10.3)	22 (14.3)	1.28 (0.39–4.18)	1.46 (0.43–4.98)	0.545
Permissive	18 (46.2)	12 (7.8)	10.59 (4.35–25.78)	10.31 (3.92–27.10)	<0.001

OR: Odds Ratio; CI: Confidence Interval; aOR: adjusted Odds Ratio. Outcome variable: anemia status. Adjusted model included child age group, maternal education, and family income. Reference categories: authoritative parenting, age 25–59 months, middle ma-ternal education, and family income. Reference categories: authoritative parenting, age 25–59 months, middle maternal education, and family income ≤ regional minimum wage.

**Table 4:** Association between anemia status and developmental delay among under-five children.

Exposure Variable	Delay *n* (%)	Normal *n* (%)	Crude POR (95% CI)	Adjusted OR (95% CI)	*p*-Value for aOR
Anemia status					
Non-anemia	8 (5.2)	146 (94.8)	Reference	Reference	—
Anemia	17 (43.6)	22 (56.4)	14.10 (5.44–36.55)	19.49 (6.46–58.84)	<0.001

Outcome variable: developmental delay. Adjusted model included child age group, maternal education, and family income.

**Table 5:** Multivariate logistic regression analysis for anemia and developmental delay.

Model/Outcome	Variable	Adjusted OR (95% CI)	*p*-Value
Anemia	Parenting style (ref: authoritative)		
	Authoritarian	1.46 (0.43–4.98)	0.545
	Permissive	10.31 (3.92–27.10)	<0.001
	Child age group (ref: 25–59 months)		
	6–12 months	3.39 (1.22–9.41)	0.019
	13–24 months	3.77 (1.43–9.97)	0.007
	Maternal education (ref: middle)		
	Low	1.71 (0.67–4.34)	0.258
	High	0.89 (0.17–4.75)	0.891
	Family income > regional minimum wage	1.97 (0.61–6.33)	0.255
Developmental delay	Anemia	19.49 (6.46–58.84)	<0.001
	Child age group (ref: 25–59 months)		
	6–12 months	0.67 (0.17–2.65)	0.570
	13–24 months	0.52 (0.14–1.93)	0.328
	Maternal education (ref: middle)		
	Low	1.15 (0.40–3.33)	0.792
	High	1.21 (0.08–18.24)	0.890
	Family income > regional minimum wage	0.21 (0.03–1.52)	0.123

aOR = adjusted odds ratio. ref = reference category. The 25–59-month age group, middle maternal education, and income ≤ regional minimum wage were used as reference categories. Because the sample included only 39 anemia events and 25 developmental delay events, adjusted estimates should be interpreted cautiously.

## Data Availability

The data presented in this study are available on request from the corresponding author due to ethical and privacy restrictions involving minors’ health and developmental information.

## Abbreviations

The following abbreviations are used in this manuscript:

AOR	Adjusted odds ratio
CBC	Complete blood count
CI	Confidence interval
Hb	Hemoglobin
IDA	Iron deficiency anemia
KPSP	Kuesioner Pra Skrining Perkembangan (Developmental Pre-Screening Questionnaire)
MCH	Mean corpuscular hemoglobin
MCHC	Mean corpuscular hemoglobin concentration
MCV	Mean corpuscular volume
MI	Mentzer index
MPASI	Complementary Feeding (Indonesian term for Infant and Young Child Complementary Feeding)
POR	Prevalence odds ratio
PSDQ	Parenting Styles and Dimensions Questionnaire
RBC	Red blood cell
Ret-He	Reticulocyte hemoglobin equivalent
SLI	Shine and Lal Index
UMK	Upah Minimum Kota (City Minimum Wage)
WHO	World Health Organization
